# Successful diagnosis of tuberculous lymphadenitis by loop-mediated isothermal amplification of cutaneous samples from an ulcerated surface lesion: a case report

**DOI:** 10.1186/1752-1947-8-254

**Published:** 2014-07-16

**Authors:** Shuichi Kawano, Takuya Maeda, Junichi Watanabe, Yuji Fujikura, Kei Mikita, Yu Hara, Soichiro Kanoh, Fumihiko Kimura, Yasushi Miyahira, Akihiko Kawana

**Affiliations:** 1 Division of Infectious Diseases and Pulmonary Medicine, Department of Internal Medicine, National Defense Medical College, 3-2, Namiki, Tokorozawa, Saitama 359-8513, Japan; 2 Division of Hematology, Department of Internal Medicine, National Defense Medical College, 3-2, Namiki, Tokorozawa, Saitama 359-8513, Japan; 3 Department of Global Infectious Diseases and Tropical Medicine, National Defense Medical College, 3-2, Namiki, Tokorozawa, Saitama 359-8513, Japan

**Keywords:** Fine-needle aspiration, Loop-mediated isothermal amplification, *Mycobacterium tuberculosis*, Tuberculous lymphadenitis

## Abstract

**Introduction:**

Tuberculous lymphadenitis is the most frequent form of extrapulmonary tuberculous. Although nucleic acid amplification assays such as polymerase chain reaction have recently become mainstream techniques for diagnosing tuberculous lymphadenitis, they are still not routinely performed in developing countries because of their high costs and complicated procedures.

**Case presentation:**

We describe a case of tuberculous lymphadenitis in a 79-year-old Japanese man who had been on continuous hemodialysis for end-stage renal disease. We employed loop-mediated isothermal amplification and the procedure for ultrarapid extraction to develop a fast and easy-to-perform procedure for diagnosing tuberculous lymphadenitis.

**Conclusions:**

The commercially available loop-mediated isothermal amplification assay kit and a rapid purification procedure enabled us to identify and amplify a *Mycobacterium tuberculosis*–specific gene within just 1.5 hours.

## Introduction

Tuberculous (TB) lymphadenitis is the most frequent form of extrapulmonary tuberculosis and cervical lymphadenopathy is its most common clinical manifestation [1]. Because lymphadenopathy can be attributed to multiple etiologies, such as malignancy, autoimmune disease and infection, it is often difficult to differentiate TB lymphadenitis from other forms of lymphadenitis on clinical grounds alone. The advent of novel diagnostic techniques, in particular loop-mediated isothermal amplification (LAMP), provides accessible, cost-effective, easy-to-perform methods for diagnosing pulmonary TB by examination of respiratory specimens [2]. Boehme *et al.* demonstrated that LAMP has a diagnostic success rate of 97.7% for smear-positive sputum specimens and 48.8% for smear-negative but culture-positive specimens [3]. In our present case, to detect *Mycobacterium tuberculosis* in a patient diagnosed with TB lymphadenitis, we examined boiled swab samples taken from an ulcerative lesion by LAMP after applying the procedure for ultrarapid extraction (PURE) to isolate target deoxyribonucleic acid (DNA) molecules for use as LAMP reagents.

## Case presentation

**Figure 1:**
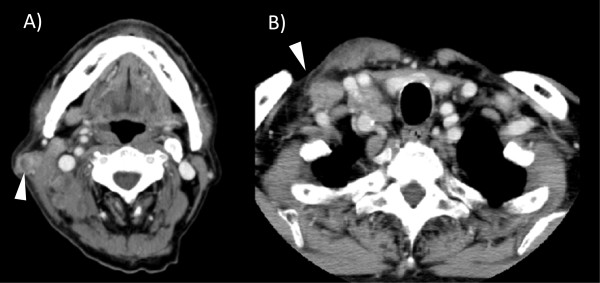
**Contrast-enhanced computed tomography scan.** Arrowheads point to multiple enlarged lymph nodes at the lateral margin of the right sternocleidomastoid muscle **(A)** and in the supraclavicular region **(B)**.

A 79-year-old Japanese man who had been on continuous hemodialysis for end-stage renal disease since 2004 visited an outpatient clinic complaining of a unilateral cervical mass and intermittent fever of more than three months’ duration. An examination at the clinic revealed three palpable right cervical lymph nodes. His body temperature was 37.4°C, with no chills or night sweats. Key laboratory data upon admission are shown in Table 1. The findings of the lab test for human immunodeficiency virus (HIV) were negative. Computed tomography of the neck and chest revealed multiple enlarged lymph nodes measuring 40mm×20mm with low central attenuation predominantly along the lateral margin of the right sternocleidomastoid muscle (Figure 1A). In addition, several enlarged lymph nodes measuring 29mm×30mm in the supraclavicular region were also noted (Figure 1B). The result of an assay for interferon γ release was positive [4].

**Table 1:** **Laboratory findings upon admission**^**a**^

**Peripheral blood**		**Blood chemistry**	
WBCs	6800/μL	T-bil	0.3mg/dL
RBCs	439×10^4^/μL	AST	14IU/L
Hb	11.4g/dL	ALT	7IU/L
Hct	37.2%	LDH	190IU/L
PLT	23.0×10^4^/μL	ALP	321IU/L
Seg	65.3%	γ-GTP	23IU/L
Eos	2.1%	TP	6.4g/dL
Ly	19.3%	BUN	20.0mg/dL
Mo	12.9%	Cr	5.65mg/dL
		CPK	19.0IU/L
		Na^+^	140mEq/L
		K^+^	3.9mEq/L
		Cl	101mEq/L
		Glc	88mg/dL
		CRP	<0.05mg/dL
		ESR	74mm/hour
		sIL-2R	2.05U/mL

^a^ALP, Alkaline phosphatase; ALT, Alanine aminotransferase; AST, Alanine aminotransferase; BUN, Blood urea nitrogen; Cl, Chloride; CPK, Creatine phosphokinase; Cr, Creatinine; CRP, C-reactive protein; Eos, Eosinophils; ESR, Erythrocyte sedimentation rate; Glc, Glucose; γ-GTP, γ-Glutamyl transpeptidase; Hb, Hemoglobin; Hct, Hematocrit; K^+^, Potassium; LDH, Lactate dehydrogenase; Ly, Lymphocytes; Mo, Monocytes; Na^+^, Sodium; PLT, Platelets; RBC, Red blood cells; Seg, Segmented neutrophils; sIL-2R, Soluble interleukin 2 receptor; T-bil, Total bilirubin; TP, Total protein; WBC, White blood cells.

Suspecting TB lymphadenitis, we performed fine-needle aspiration (FNA) of the para-sternocleidomastoid nodes. The obtained specimens were examined by microscopy (smears for acid-fast bacilli and fungi), microbiological culture and cytology. The smears were negative for acid-fast bacilli, and the cytology was determined to be class II, partly due to the presence of a mass of neutrophils. Among the diagnostic procedures we used, only the six-week culture for tuberculosis with the BacT/ALERT® three-dimensional microbial detection system (bioMérieux, Nürtingen, Germany) was positive, and *M. tuberculosis* DNA was unequivocally identified in the positive culture by means of the COBAS® TaqMan® MTB assay (Roche Diagnostics, Rotkreuz, Switzerland) [5].

**Figure 2:**
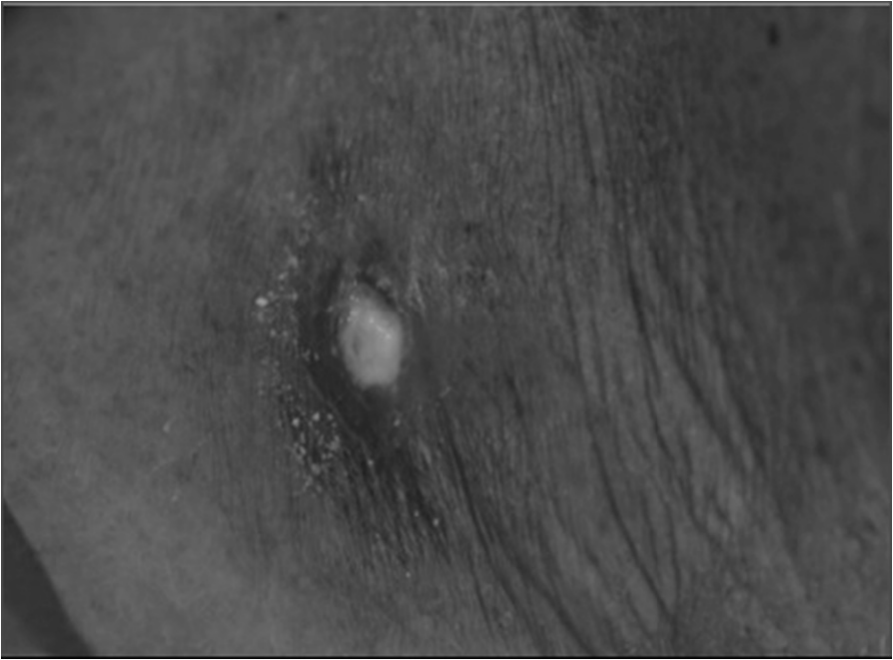
Photograph showing an ulcerated lesion on the right side of the neck.

**Figure 3:**
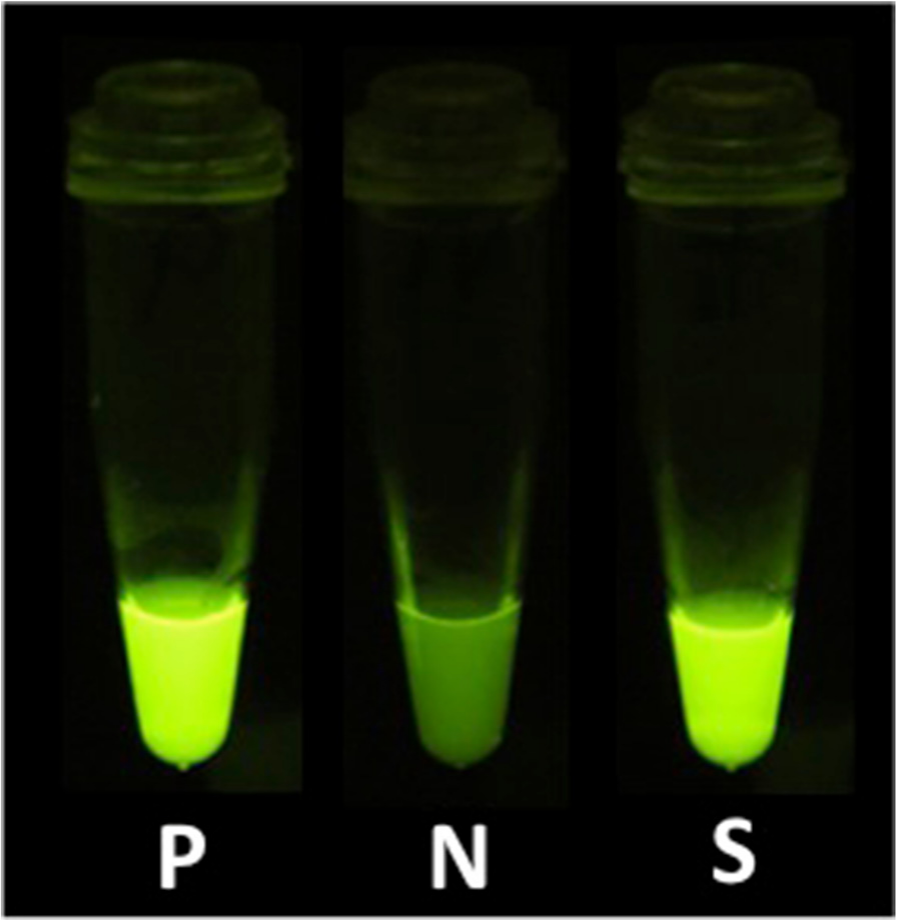
**Detection of *Mycobacterium tuberculosis *by loop-mediated isothermal amplification assay showing the color change to cloudy yellow.** N: negative control; P: positive control; S: clinical sample.

In this case, we reached a definitive diagnosis of TB lymphadenitis in an efficient manner by using a commercially available LAMP kit (Eiken Chemical, Tokyo, Japan) to amplify an *M. tuberculosis*-specific gene and a PURE kit (Eiken Chemical) to isolate the DNA target of interest [6]. Cutaneous swab samples were obtained by wiping the surface of an ulcerated lesion (Figure 2) with a sterile cotton swab and placing it into 960μL of DNA extraction solution. The suspension was incubated on a heat block at 100°C for 10 minutes, and DNA was rapidly extracted with the PURE kit in 30μL of solution [6]. Amplification of the *M. tuberculosis–*specific gene was achieved by LAMP at 64°C for 40 minutes according to the manufacturer’s instructions, and a positive LAMP result was indicated by the color change observed under ultraviolet light (Figure 3). On the basis of these results, we diagnosed TB lymphadenitis.

The patient subsequently received anti-tuberculosis chemotherapy consisting of isonicotinic acid hydrazide (300mg/day), rifampicin (450mg/day) and ethambutol hydrochloride (500mg/day after dialysis). Two weeks later, he became afebrile and had no signs of exacerbation, and his painful lymphadenopathy eventually subsided.

## Discussion

The current gold standard methods for diagnosing tuberculosis are microscopy and culture. However, the poor sensitivity of microscopy and the laborious, time-consuming procedures of culturing make these modalities less than ideal. Therefore, nucleic acid amplification assays such as polymerase chain reaction (PCR) have recently become mainstream techniques for diagnosing tuberculosis [7]. However, the molecular biological technologies on which such methods are based require not only experience and expertise but also expensive equipment; this is the principal reason why most of these sophisticated diagnostic tools are not suitable for use in resource-limited, disease-endemic regions. In contrast, LAMP can amplify *M. tuberculosis*–specific DNA rapidly under isothermal conditions [8]. A recent meta-analysis of 10 studies conducted to estimate the diagnostic accuracy of LAMP for pulmonary tuberculosis revealed a sensitivity of 80.0% (95% confidence interval (CI), 78%–83%) and a specificity of 96.0% (95% CI, 95.0%–97.0%) [9]. Furthermore, LAMP, which, in combination with PURE, has recently been established as a diagnostic technique requiring only limited equipment and manpower, provides an accessible, cost-effective, rapid, and more appropriate molecular diagnostic tool for tuberculosis in the field setting [6]. All procedures, including the time required for sample preparation, can be performed within just 1.5 hours.

FNA is an appropriate procedure to use in the initial evaluation in diagnostic methods targeting cervical nodular lesions, including TB lymphadenitis [10,11]. Although FNA is a non-invasive, inexpensive, viable alternative to excisional biopsy of the lymph nodes, the detection rate of *M. tuberculosis* by examining aspirate specimens is disappointingly low compared to microbiological techniques. Some researchers have suggested that a combination of FNA and PCR might increase the sensitivity for diagnosing TB lymphadenitis [12]. However, the overall sensitivity of PCR with aspirate specimens is significantly lower than that with biopsy specimens and histopathological examinations [13]. PCR is also notorious for yielding false-negative results. It is thus important to establish a rapid, highly sensitive molecular diagnostic method for use with FNA specimens before proceeding to more invasive procedures such as additional excisional biopsy of the lymph nodes. Although excisional biopsy for microbiological, molecular biological and histopathological evaluations has the highest diagnostic yield [11,14], it is also the most invasive means of reaching a diagnosis. LAMP, on the other hand, is a rapid diagnostic procedure that also plays an important role in deciding which specimen-collecting procedure to employ.

## Conclusions

LAMP combined with PURE enabled us to detect *M. tuberculosis* from swabbed samples taken from an ulcerative lesion that had purulent exudate from the lymph nodes. The procedure could be performed in just 1.5 hours, making it beneficial and convenient for use in resource-limited, disease-endemic regions. We believe that the new commercial LAMP kit not only will be useful as a primary testing tool for TB but also will provide additional diagnostic value for TB lymphadenitis. Further studies are necessary to evaluate the sensitivity and specificity of this diagnostic tool.

## Consent

The Ethics Committee of the National Defense Medical College Research approved the design and protocol of this study (reference no. 1205). Written informed consent was obtained from the patient for publication of this case report and any accompanying images. A copy of the written consent is available for review by the Editor-in Chief of this journal.

## Abbreviations

FNA: Fine-needle aspiration; LAMP: Loop-mediated isothermal amplification; PCR: Polymerase chain reaction; PURE: Procedure for ultrarapid extraction; TB: Tuberculous.

## Competing interests

The authors declare that they have no competing interests.

## Authors’ contributions

Skaw conceived the study, participated in its design and coordination and helped draft the manuscript. TM was directly involved in the overall care of the patient and drafted the manuscript. JW, YF, KM, YH, SKan, FK, YM and AK were involved in data investigation and the patient’s treatment. All authors read and approved the final manuscript.
